# The Role of the Aryl Hydrocarbon Receptor in Vascular Factors Related to Preeclampsia in a Smoking Mouse Model

**DOI:** 10.3390/cimb46010048

**Published:** 2024-01-16

**Authors:** Ho-Yeon Kim, Ye-Seon Seok, Hye-Yeon Moon, Geum-Joon Cho, Ki-Hoon Ahn, Soon-Cheol Hong, Min-Jeong Oh, Hai-Joong Kim

**Affiliations:** 1 Department of Obstetrics and Gynecology, Korea University School of Medicine, Seoul 02841, Republic of Korea; shinbi73@korea.ac.kr (H.-Y.K.); jjmoon58@naver.com (H.-Y.M.); akh1220@korea.ac.kr (K.-H.A.); novak082@naver.com (S.-C.H.); haijkim@korea.ac.kr (H.-J.K.); 2 Department of Obstetric and Gynecology, Korea University Guro Hospital, Seoul 08308, Republic of Korea; yeseon@korea.ac.kr

**Keywords:** aryl hydrocarbon receptor, soluble FMS-like tyrosine kinase, cigarette

## Abstract

Smoking cigarettes is known to lower the risk of preeclampsia. The objective of this study is to evaluate the effect of smoking on the expression of soluble FMS-like tyrosine kinase-1 (sFlt-1), vascular endothelial growth factor (VEGF), and endoglin (sEng)-1 and the role of the aryl hydrocarbon receptor (AhR) in pregnant mice. We developed a smoking mouse model using a gas-filling system. One or two cigarettes per day were exposed to each of the five pregnant mice for five days a week throughout pregnancy. AhR agonist and antagonist were injected. Serum levels and expression in the placenta of sFlt-1, VEGF, and sEng-1 were analyzed and compared among the cigarette smoke and no-exposure groups after delivery. Compared to the no-smoke exposure group, the serum level of sFlt-1 was significantly decreased in the two-cigarette-exposed group (*p* < 0.001). When the AhR antagonist was added to the two-cigarette-exposed group, sFlt-1 levels were significantly increased compared to the two-cigarette group (*p* = 0.002). The levels of sFlt-1 in the AhR antagonist group did not change regardless of two-cigarette exposure (*p* = 0.064). With the AhR agonist, sFlt-1 decreased significantly compared to the control (*p* = 0.001) and AhR antagonist group (*p* = 0.002). The sFlt-1 level was significantly decreased after the injection of the AhR agonist compared to the control group (*p* = 0.001). Serum levels of VEGF were significantly decreased in the one-cigarette-exposed group compared to the control group; however, there was no difference between the control and the two-cigarette-exposed groups. The placental expression of sFlt-1, VEGF, and sEng were inconsistent. This study offers insights into the potential role of AhR on antiangiogenic sFlt-1 associated with preeclampsia. It may support the invention of a new treatment strategy for preeclampsia using AhR activation.

## 1. Introduction

Preeclampsia is a devastating obstetric disease that causes complications in 4–8% of pregnancies. Every year, 10,000 women suffer from preeclampsia in South Korea [1]. The diagnosis of preeclampsia includes high blood pressure and proteinuria after 20 weeks of gestation [2]. An enormous amount of research conducted on this condition has not yet identified the etiology of preeclampsia, and the only cure is the delivery of the fetus. However, the shallow invasion of the endovascular trophoblast during implantation and the endothelial dysfunction caused by free-circulating radicals secreted from the placenta with low oxygenation are possible pathogeneses. In addition, the overwhelming antiangiogenic property over proangiogenesis is an essential mechanism in preeclampsia.

Risk factors for the development of preeclampsia have been extensively investigated, including primiparity, being over 35 years of age, multifetal gestation, a previous history of preeclampsia, chronic hypertension, obesity, diabetes mellitus, and vascular and connective tissue disorders [3]. However, there is one factor that appears to lower the incidence of preeclampsia. Smoking during pregnancy is one lifestyle factor that has demonstrated a protective role in many epidemiologic studies [4,5,6]. One meta-analysis revealed a 32% lower incidence of preeclampsia in pregnant women who smoke [7]. Although the mechanism involved in reducing the effect of preeclampsia with smoking is uncertain, nicotine might play a role that selectively inhibits thromboxane A2 via the inhibition of thromboxane synthase, where pathophysiology indicates increased thromboxane A2 production in preeclampsia [8,9]. Another theory states that nicotine receptor activation in the placenta stimulates nitric oxide and thereby prevents preeclampsia. Alternatively, both mechanisms concurrently take place [10]. The inhibition of cytokine production and activation of the antioxidant property via nicotine can lead to the beneficial effect of smoking, evidenced both in vitro and in vivo [11,12]. Since there are over 4000 components in tobacco smoke, most research has focused on nicotine, making it challenging to pinpoint exactly what influences the development of preeclampsia.

Cigarette smoking influences the human body using the aryl hydrocarbon receptor (AhR) known as the dioxin receptor, which binds the components of cigarette smoke such as benzopyrene and nitrosamine [13]. After binding, these compounds enter the nucleus and are involved in transcription. AhR is a ligand-activated transcription factor that was first recognized as a modulator of exogenous chemicals, and recent findings have elucidated the various roles of this factor, especially for immune and inflammatory responses [14,15] and the maintenance of pregnancy [16,17]. Fetal organ epithelium, the endothelium of vessels, and syncytiotrophoblasts abundantly express AhR, which have important roles in trophoblast invasion and the reduction of oxidative stress [18,19]. Since smoking has a protective role in preeclampsia, there seems to be a link between the abundant expression of AhR in the placenta and its role in preeclampsia. One previous theory stated that the underlying mechanism of preeclampsia, which is a shallow placentation followed by abnormal arteriole remodeling, is compensated by the activation of AhR with smoking [20]. The activation of AhR could lead to enhanced trophoblastic invasion and alleviate preeclampsia.

Important antiangiogenic factors that influence preeclampsia and are significantly increased in this condition are sFlt-1 and soluble endoglin (sEng). It has been postulated that the imbalance between proangiogenic and antiangiogenic factors may cause or be the consequence of this disease, although these antiangiogenic factors are preceded by increases in serum levels several weeks before the clinical onset of the disease. Animals with high sFlt-1 and sEng have been shown to display severe preeclampsia features [21]. Several investigations have connected angiogenic factors and the constituents of smoke. In this clinical study, the level of soluble FMS-like tyrosine kinase-1 (sFlt-1) in a non-pregnant smoker was observed to be lower than that of a non-smoker [22]. In a human observational study, women with preeclampsia who smoked before or smoked during pregnancy favored pro-angiogenic states throughout the three trimesters [23]. One study using human placental tissues with exposure to cigarette smoke demonstrated reduced sFlt-1 and abundant PlGF [24]. Several other in vitro studies have shown reduced levels of sFlt-1 and elevated placental growth factor (PlGF) in subjects exposed to smoking and the progression of the proangiogenic state in trophoblasts [25,26]. The protective effect of smoking was explained partly by carbon monoxide’s effect on reducing apoptosis; however, after the cessation of smoking, the protective effect on preeclampsia remained [27]. There may be other components of cigarette smoke that alleviate the preeclampsia phenomenon, such as AhR activation.

Studies evaluating the relationship between angiogenic markers related to preeclampsia and cigarette smoking are limited. Understanding the biological mechanisms and individual components of smoking that may protect against preeclampsia may help to elucidate the pathogenesis of this obstetric complication and contribute to determining treatment or preventive strategies. Here, we show the expression of different angiogenic factors related to preeclampsia under the influence of cigarette smoke and the role of the AhR utilizing a smoking mouse model.

## 2. Materials and Methods

### 2.1. Animals

CD-1 outbred mice were purchased from Charles River Laboratories (Wilmington, MA, USA) and maintained under a 14 h light/10 h dark illumination at 23 °C with food and water available ad libitum. Experimental pregnant mice were obtained after mating. Day 1 of gestation (E1) was designated the first day after observing the vaginal plug, which was formed by secretions from the male vesicular and coagulating glands, and is a convenient and easily visible indicator that mating has occurred. Coeval pregnant mice were used to check the health conditions during all stages of the experiment. All animal procedures were approved by the Korea University Animal Ethics Committee (KUIACUC-2012-121, 30 March 2012).

### 2.2. Exposure to Cigarette Smoke

Pregnant mice were randomly divided into four groups. The non-exposed group comprised control pregnant mice that received no intervention; the one-cigarette-exposed group comprised pregnant mice that were exposed individually to one cigarette of smoking/day during pregnancy; and the two-cigarette-exposed group comprised pregnant mice that were exposed individually to two cigarettes of smoking/day during pregnancy. Five animals were studied in each group. Pregnant mice in the one-cigarette and two-cigarette groups were exposed to one or two cigarettes a day for 5 days/week, respectively (code 3R4F reference cigarettes, produced for the University of Kentucky Tobacco and Health Research Institute, Lexington, KY, USA), from E1 to delivery. On each day of exposure, animals were placed individually inside a Plexiglas cabinet (40 × 90 × 100 mm). Cigarette smoke was delivered into the cabinet via air inflow at a rate of 1.7 mL/s from a burning cigarette in the chamber, and the combustion time of the cigarette was less than 3 min. This exposure amount was equivalent to ~3–4 packs/day in humans [19]. A ventilator inside the cabinet ensured the rapid and equal distribution of smoke. Fresh air was delivered into the cabinet to remove the smoke. The control, non-exposed group was placed in an identical cabinet for the same period, and these mice were not exposed to smoke.

### 2.3. Aryl Hydrocarbon Receptor (AhR) Agonist and Antagonist

The AhR agonist (Ficz, Enzo, NY, USA) and antagonist(were injected intraperitoneally in three different groups for 5 days/week from E1 to delivery. Pregnant mice in the AhR agonist and antagonist groups received 1 µg/day/mouse of the AhR agonist (6-formylindolo(3,2-b) carbazole) and 10 µg/day/mouse of the AhR antagonist (6,2′4′-trimethoxyflavone, USA, TOCRIS), respectively. The third group received 10 µg/day/mouse of the AhR antagonist and was exposed to 2 cigarettes a day for 5 days/week.

### 2.4. Collection of Blood and Placenta Samples

After exposure, each mouse was anesthetized and sacrificed to obtain placental tissues and blood samples. Blood samples were centrifuged at 1000× *g* for 10 min at 4 °C. The supernatant was collected as serum and stored at −70 °C.

### 2.5. Assays

The measurement of sFlt-1, sEng, and the vascular endothelial growth factor (VEGF) were performed with a commercially available sandwich enzyme-linked immunosorbent assay kit (Quantikine ELISA kit; R&D Systems, Minneapolis, MN, USA). The volume of media used was determined using pilot assays and ranged from 3.8 to 15.2 pg/mL for sFlt-1 and 1.28 to 13.6 pg/mL for sEng. The minimum detectable dose of mouse VEGF was typically less than 3.0 pg/mL. Media were assayed in duplicate for all experiments.

### 2.6. Western Blotting

Placental tissues were unfrozen and lysed in a protein lysate buffer (50 mM HEPES (pH 7.5) with 150 mM of NaCl, 1.5 mM of MgCl_2_, 1 mM of ethylenediaminetetraacetic acid, 10% glycerol, 1% Triton X-100, and protease inhibitors (aprotinin, phenylmethylsulfonyl fluoride, and sodium orthovanadate). Using a rotor-stator homogenizer, tissues were homogenized, centrifuged (12,000 rpm, 15 min, 4 °C), and quantified with the Bradford reagent. The same amount of each sample was prepared and boiled at 95 °C for 5 min in a 4× sodium dodecyl sulfate loading buffer. Electrophoresis was performed in 12% acrylamide gel and transferred to a Nytran-plus membrane (pore size: 0.45 µm, Amersham Pharmacia Biotech, Piscataway, NJ, USA) using a 1× transfer buffer (39 mM glycine, 48 mM of Tris base, 0.037% sodium dodecyl sulfate, and 20% methanol). The transferred membrane was cleansed in a blocking buffer solution (5% non-fat dried milk and 0.02% sodium azide) followed by incubation with primary anti-rabbit VEGF (1:500) (Santa Cruz Biotechnology, Santa Cruz, CA, USA) and primary anti-rabbit sFlt-1 (1:1000, Abcam, Waltham, MA, USA) at 4 °C for 24 h. This was followed by cleansing with a 1× phosphate-buffered saline solution and NaCl/Tris-Cl. Secondary anti-rabbit GAPDH (1:2000) (Bio-Rad Laboratories, Hercules, CA, USA) was incubated for 2 h, and the samples were visualized using the West Dura Extended Duration Substrate (Pierce Chemical Co., Dallas, TX, USA).

### 2.7. Immunocytochemistry

Mouse placentas were fixed with paraformaldehyde, permeabilized with 0.02% Tripton X-100, and blocked with 3% BSA for 45 min. Samples were incubated with anti-mouse sFlt-1 (1:50 in 5% BSA, Abcam, Waltham, MA, USA) and anti-rabbit VEGF (1:100 in 5% BSA, Abcam, Waltham, MA, USA) overnight at 4 °C. After washing, an Alexa-488-conjugated anti-mouse IgG (1:500, Invitrogen, Carlsbad, CA, USA) was used as the secondary antibody. Nuclei were counterstained with Hoechst 33342 (1:5000, Invitrogen) for immunofluorescence and were labeled in blue. Confocal microscopy (LSM 700; Zeiss, Wetzlar, Germany) was used for the morphologic evaluation.

### 2.8. Statistical Analysis

Statistical analysis was performed with SPSS ver. 20.0 (SPSS Inc., Chicago, IL, USA). Normality distribution was assessed using the Shapiro–Wilk method. Data are presented as the mean ± standard deviation for biochemical data. ANOVA was used for the comparison of continuous variables, and Tukey’s method was used for post hoc analysis. Differences were considered statistically significant at *p* < 0.05.

## 3. Results

### 3.1. Comparison of Serum sFlt-1 of Mice Exposed to Cigarette Smoking

**Figure 1:**
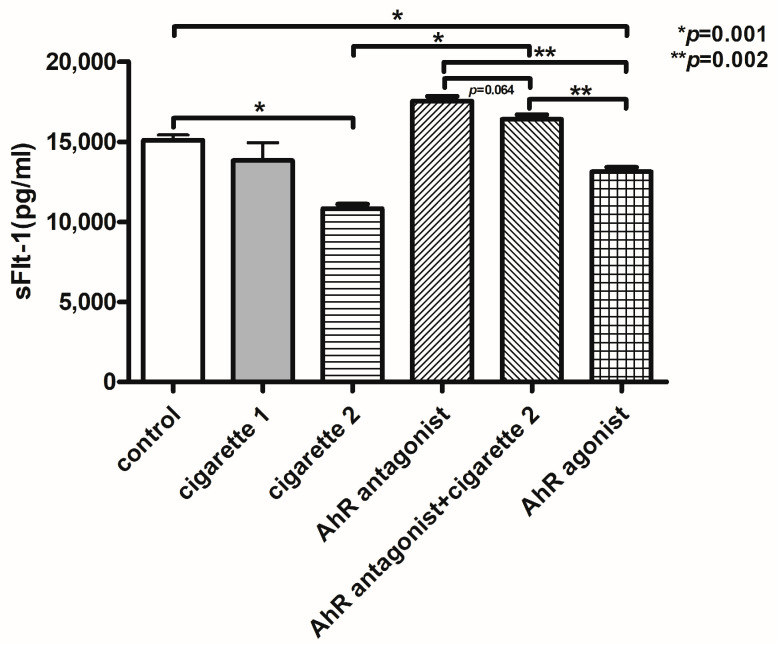
ELISA results of serum soluble FMS-like tyrosine kinase-1 (sFlt-1) in various groups of mice exposed to different levels of cigarette smoke and treatment with the Aryl hydrocarbon receptor antagonist and agonist. The duplication of all assays was conducted. All values are the mean ± SD.

**Figure 2:**
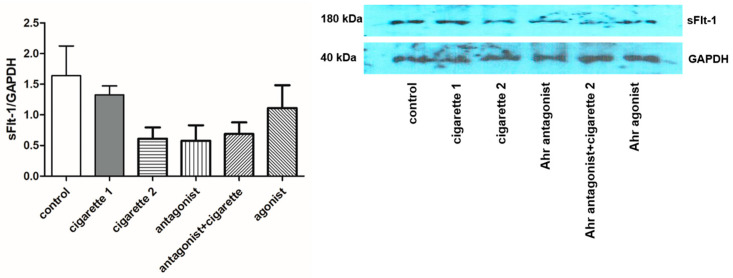
Expression of placenta soluble FMS-like tyrosine kinase-1 (sFlt-1) in Western blot analysis. Six independent blots and forms of staining were conducted. All values are the mean ± SD. sFlt-1 indicates soluble FMS-like tyrosine kinase-1.

**Figure 3:**
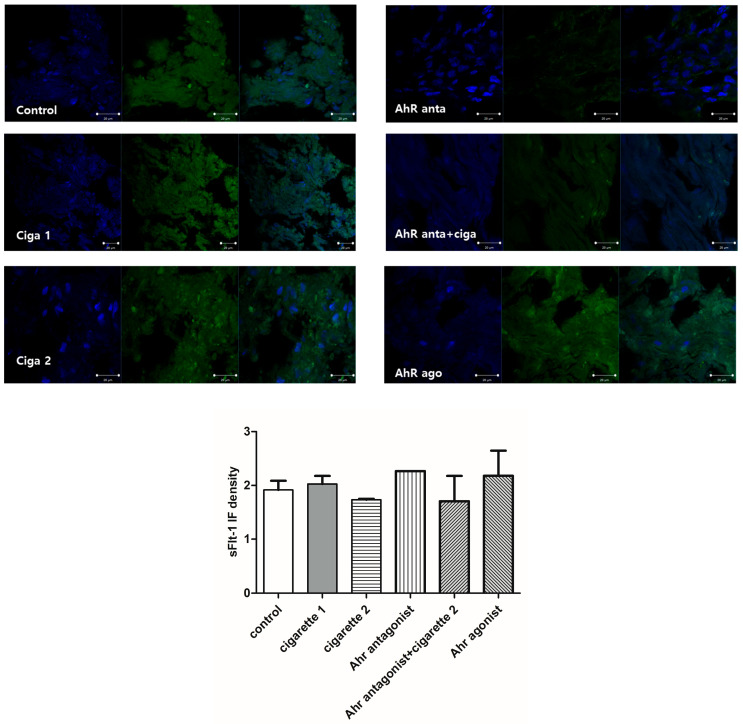
Immunofluorescent staining for sFlt-1 in the placenta. FITC (Green), DAPI (blue).

Compared to the control group, the serum level of sFlt-1 was significantly decreased in the two-cigarette exposure group (*p* < 0.001) (Table 1, Figure 1). All mice exposed to three cigarettes per day experienced aborted fetuses. Adding the AhR antagonist slightly increased sFlt-1. When the AhR antagonist was added to the two-cigarette-exposed group, sFlt-1 levels were significantly increased compared to the two-cigarette group (*p* = 0.002). The levels of sFlt-1 in the AhR antagonist group did not change regardless of two-cigarette exposure (*p* = 0.064). With the AhR agonist, sFlt-1 decreased significantly compared to the control (*p* = 0.001) and AhR antagonist group (*p* = 0.002). However, the sFlt-1 expression in the placental tissue using Western blotting showed no differences among the groups, while a slightly decreased expression was observed in the AhR antagonist and AhR+2-cigarette group (Figure 2). In immunofluorescent staining, sFlt-1 expression was slightly attenuated with two cigarettes compared to the control and one cigarette. Adding the AhR antagonist with two cigarettes showed a slightly decreased expression of sFlt-1, but the AhR antagonist and agonist did not influence the placental expression of sFlt-1 (Figure 3).

### 3.2. Vascular Endothelial Growth Factor (VEGF)

**Figure 4:**
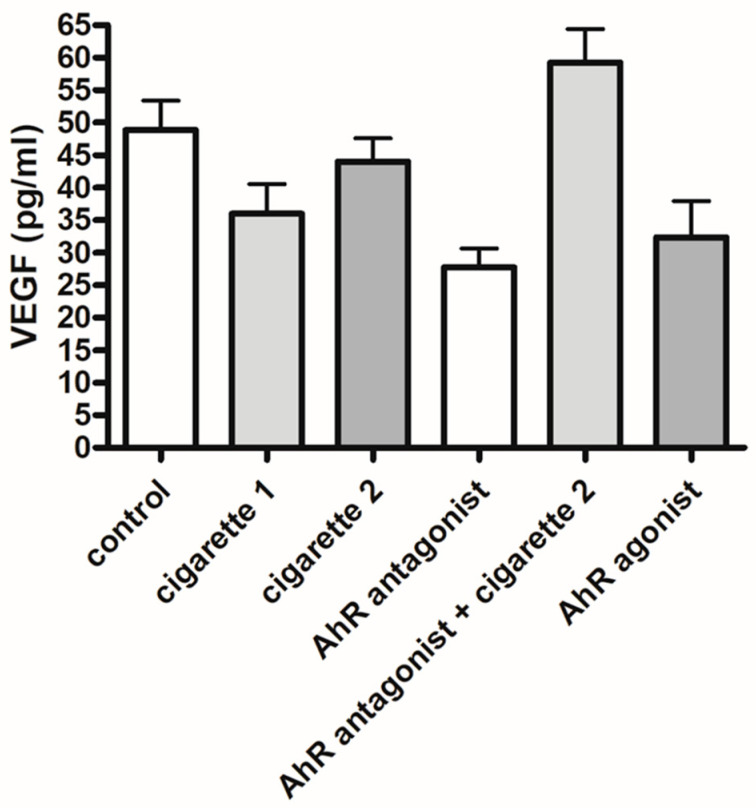
ELISA results of serum vascular endothelial growth factor (VEGF). The duplication of all assays was conducted. All values are the mean ± SD.

**Figure 5:**
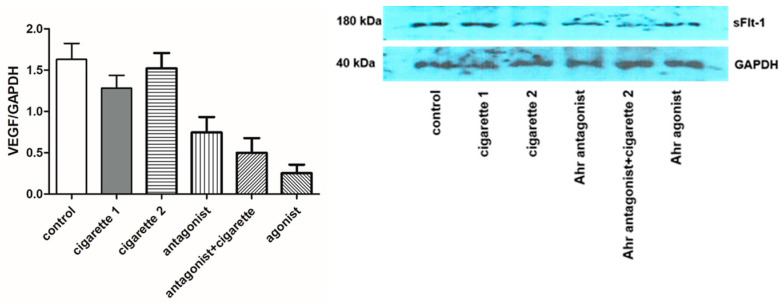
Expression of placenta vascular endothelial growth factor (VEGF) in Western blot. Three independent blots and forms of staining were conducted. All values are the mean ± SD.

**Figure 6:**
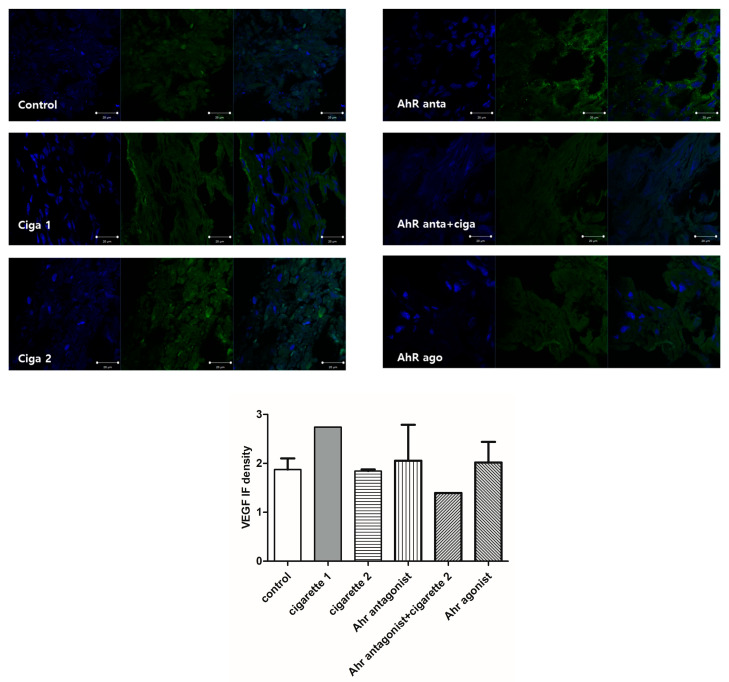
Immunofluorescent staining for VEGF in the placenta. FITC (Green), DAPI (blue).

The serum levels of VEGF were significantly decreased in the one-cigarette exposure group compared to the control group; however, there was no difference between the control and the two-cigarette-exposed groups (Figure 4). The serum levels of VEGF in the two-cigarette-exposed group were elevated compared to the one-cigarette group. With the AhR antagonist and agonist, VEGF levels decreased but not significantly. In the Western blotting analysis and immunofluorescent staining, no significant differences in VEGF expression were observed among the groups (Figure 5 and Figure 6). When the AhR antagonist or AhR agonist was added, VEGF expression decreased but not significantly in Western blotting. VEGF expression in immunocytochemistry showed inconsistent results among the groups.

### 3.3. Soluble Endoglin (sEng)

**Figure 7:**
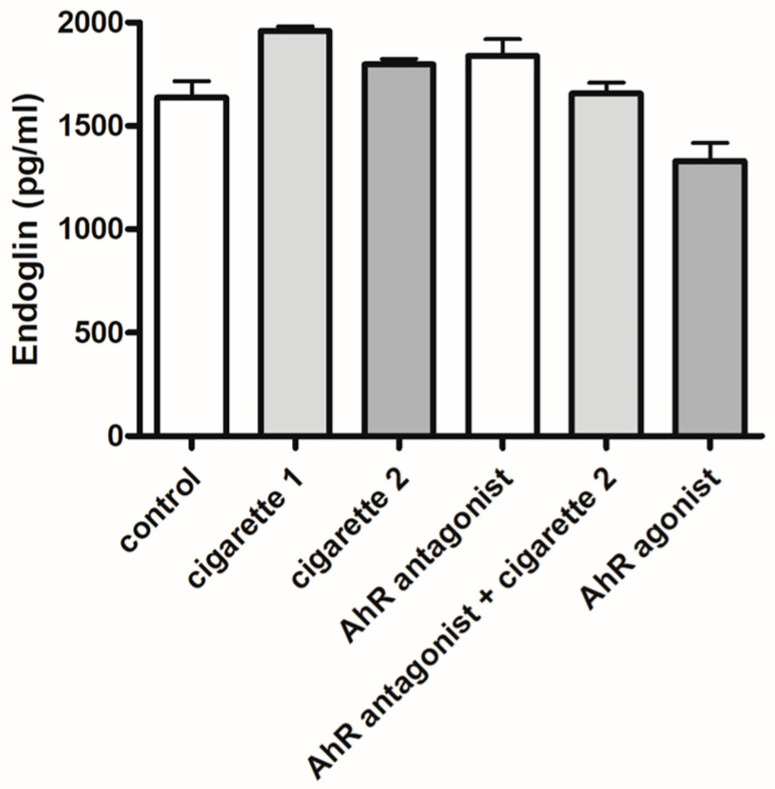
ELISA results of serum soluble endoglin (sEng). The duplication of all assays was conducted. All values are the mean ± SD.

There were no differences in the serum levels of sEng among the one- or two-cigarette-exposed groups and the control group. The addition of the AhR antagonist did not influence serum levels of sEng but showed a decreasing trend (Figure 7).

### 3.4. AhR Agonist and Antagonist and sFlt-1

sFlt-1 levels did not decrease when the AhR antagonist was administered in the two-cigarette-exposed group, and, compared to the AhR antagonist-treated group without cigarettes, the sFlt-1 level was not different (*p* = 0.064). The sFlt-1 level was significantly decreased after the injection of the AhR agonist compared to the control group (*p* = 0.001) and the AhR antagonist-treated group (*p* = 0.002). There was a significant difference in the sFlt-1 level between the AhR agonist and AhR antagonist plus the two-cigarette-exposed group (*p* = 0.002) (Figure 1).

## 4. Discussion

Our study demonstrates that cigarette smoke reduced serum sFlt-1 levels in mice via the AhR. Decreased serum sFlt-1 levels after exposure to two cigarettes were not compensated by the addition of the AhR antagonist. The serum sFlt-1 levels were significantly elevated after two cigarettes with the AhR antagonist compared to exposure to two cigarettes only. Therefore, without AhR, i.e., in the presence of an antagonist, there was no decrease in the sFlt-1 level; thus, no beneficial effect of cigarette smoke was observed. In addition, the AhR agonist decreased the serum sFlt-1 level. The activation of AhR might have a similar effect of risk reduction on preeclampsia. This relationship between the role of sFlt-1 and AhR aided in understanding why smoking decreased the incidence of preeclampsia after exposure to cigarette smoke.

Smoking is a risk factor associated with many diseases, such as cancer and cardiovascular disease, and other obstetric complications, such as placenta abruption, fetal growth restriction, and preterm birth [28,29,30,31,32]. However, it has been known to be protective against pregnancy-induced hypertension. There are over 5000 chemical compounds in cigarette smoke, with nicotine being a major component, and its role is not fully elucidated in the obstetric field. Previous studies have shown that nicotine-facilitated endothelial proliferation lowered the sFlt-1 concentration, prevented endothelial dysfunction, and promoted hypoxia-induced VEGF secretion in human trophoblast cells [24,33,34,35]. However, Genbacev et al. [36] demonstrated that nicotine was not responsible for lowering the incidence of preeclampsia in studies involving in vivo smoking exposure. Carbon monoxide and oxidant gases are other major constituents in cigarette smoke that are potential contributors to vascular conditions [37].

Herein, we focused on AhR to define the role of smoking in pregnancy and preeclampsia. AhR, which heterodimerizes with the AhR nuclear translocator, is known as a cytosolic dioxin receptor and one of the transcription factors. It is expressed abundantly in the lungs, placenta, spleen, and ovaries [38,39]. Wakx et al. also demonstrated AhR activity and expression via endogenous ligands in the nucleus of placental cells throughout pregnancy [18]. After ligand binding in the cytoplasm, it moves to the nucleus, and this ligand-bound AhR-AhR nuclear translocator heterodimer then binds to xenobiotic-responsive elements in the promoter regions of genes involved in metabolizing carcinogens, pollutants, environmental contaminants, and drugs, or activating procarcinogens [40]. The highest exposure to AhR ligands comes from diet and cigarette smoke. Polycyclic aromatic hydrocarbons (PAHs) may be the major constituents responsible for the activation of AhR via cigarette smoke; however, other components, including dioxins, are known to activate AhR [37,41]. Specific AhR ligands were not identified in the placenta; however, they control angiogenic activity that prevents excessive angiogenesis in tissues. Not only does AhR have a function in the detoxification process, but numerous research demonstrate its role in immune response, cell cycle, metabolism, tumor proliferation, and reproduction. In experiments on AhR-knockout animals, fertility was impaired, and deformity was formed [42]; therefore, it has been stated that AhR is indispensable for maintaining pregnancy.

There have been several studies that have presented an increased expression of VEGF via AhR [43,44] and shown the protective role of AhR on lung cells through an antioxidant defense [45]; however, AhR role’s on sFlt-1 is not explained in an up-to-date way. One of the essential markers involved in preeclampsia development is sFlt-1. Many studies on preeclampsia have revealed the important role of sFlt-1, which is produced in the placenta and acts as an anti-angiogenic. sFlt-1 is a splice variant of VEGF receptor 1 and antagonizes VEGF and PlGF [46,47]. During pregnancy, sFlt-1 levels are constant until the end of the second trimester and increase until reaching a peak at 40 weeks of gestation. Abnormally high levels of sFlt-1 have been implicated in the pathogenesis of preeclampsia, and the level of sFlt-1 is usually elevated 5 weeks prior to the appearance of preeclampsia [48].

The AhR signaling pathway has been known to regulate the endothelial angiogenic activities of either antiangiogenic or proangiogenic properties [49]. Our result also demonstrated the decreasing trend of VEGF with the AhR antagonist and agonist in the placenta, implying AhR angiogenic roles. Whether and how AhR affects placental cells is elusive, either of the physiologic or pathologic state. But it is likely that cigarette smoking activates AhR in the trophoblast or endothelium in the placenta and leads to an angiogenic state by decreasing sFlt-1. This angiogenic state can facilitate normal placentation. This phenomenon observed in our study might explain why cigarette smoking prevents pregnancy-induced hypertension. We investigated for the first time the relationship between AhR and sFlt-1 and found that, without AhR, the protective role of cigarette smoking via lowering the antiangiogenic factor is absent.

Another important angiogenic factor in preeclampsia is VEGF. Recent findings have revealed that VEGF-A expression is greatly decreased in the placentas of women with preeclampsia [50,51]. However, maternal smoking greatly enhances the expression of the hypoxia-inducible factor and VEGF ligands, which are important regulators of cytotrophoblast survival and differentiation during uterine invasion [36]. This explains the beneficial effects of cigarette smoke from the stimulation of placental VEGF expression. Although our results demonstrated no differences in VEGF expression in the serum and placenta, the two-cigarette-exposed group showed an elevated trend of VEGF expression compared to the one-cigarette-exposed group. This coincides with the dose-responsive stimulatory effect on VEGF by cigarette smoke. An insufficient number of mice might be the reason for there being no observed difference in VEGF between the cigarette smoking groups and the control group. While we may not have a complete understanding of the effects of AhR on sFlt-1, VEGF, and sEng in the blood and placenta, it is evident that there are differences in the expression of these factors in the placenta related to AhR. This appears to be in line with the previous literature, which confirmed AhR’s characteristic of being differentially selective agonists or antagonists in various organs.

Smoking can lead to serious obstetric complications such as low body weight and premature placental abruption. However, according to existing research, the increase in apoptosis and oxidative stress in the lungs due to smoking can be somewhat protected by the antioxidant effects of components like catechins found in green tea [52]. Therefore, it is speculated that mitigating the adverse effects of smoking with ingredients such as green tea catechins while simultaneously stimulating the AhR action of smoking could ultimately contribute to finding a method to treat conditions like preeclampsia during pregnancy.

To our knowledge, this is the first in vivo study to define the relationship between AhR and sFlt-1 using mice. However, there are several limitations. First, we did not observe the cell signaling pathways, molecular biology, or mechanisms underlying angiogenesis to fully explain our results and only focused on single AhR activation; therefore, further research is warranted. Second, we did not observe the effect of cigarette smoke on angiogenic VEGF and antiangiogenic sEng, which are other important angiogenic factors besides sFlt-1. Third, Western blotting results were inconsistent and did not demonstrate the relationship between AhR and sFlt-1. Lastly, we did not observe a dose-dependent effect or long-term effect of prenatal cigarette smoke; thus, various doses of cigarette smoke and their long-term follow-up should be investigated in future studies.

## 5. Conclusions

Cigarette smoking during pregnancy has adverse effects, including spontaneous abortion, low birth weight, perinatal mortality, malformations, and long-term adverse effects on children. However, beneficial effects are observed in the development of preeclampsia in women who smoke. There are over a thousand constituents in cigarette smoke and numerous biological pathways by which these substances act. One of these pathways is the activation of AhR, and our results show the role of AhR on sFlt-1, which is an essential antiangiogenic factor associated with preeclampsia. Understanding how an exogenous agent activates factors that may reduce the occurrence of preeclampsia could contribute to improving the knowledge of this disease. In addition, it may be possible to invent a new treatment strategy for preeclampsia using AhR activation.

## Figures and Tables

**Table 1:** ELISA results of serum soluble FMS-like tyrosine kinase-1 (sFlt-1), the vascular endothelial growth factor (VEGF), and soluble endoglin (sEng), and the addition of the Aryl hydrocarbon receptor (AhR) antagonist and agonist. All values are the mean ± SD.

	sFlt-1 (pg/mL)	VEGF (pg/mL)	sEng (pg/mL)
control	15,096 ± 884	48.9 ± 11.9	1638 ± 204
cigarette 1	13,829 ± 2943	44.5 ± 7.6	1959 ± 55
cigarette 2	10,819 ± 743 *	43.9 ± 9.5	1797 ± 69
cigarette 3	406 ± 169	12.9 ± 5.6	1740 ± 107
AhR antagonist	17,534 ± 819 **	27.7 ± 7.6	1839 ± 211
AhR antagonist+cigarette 2	16,404 ± 767 **	59.3 ± 13.5	1657 ± 137
AhR agonist	13,117 ± 779 *	32.3 ± 14.8	1331 ± 227

AhR, aryl hydrocarbon receptor; * *p* = 0.001; compared to control; ** *p* = 0.002; compared to AhR agonist.

## Data Availability

All data are available on reasonable request to the corresponding author.
